# A Bluetooth-Enabled Electrochemical Platform Based on *Saccharomyces cerevisiae* Yeast Cells for Copper Detection

**DOI:** 10.3390/bios15090583

**Published:** 2025-09-05

**Authors:** Ehtisham Wahid, Ohiemi Benjamin Ocheja, Antonello Longo, Enrico Marsili, Massimo Trotta, Matteo Grattieri, Cataldo Guaragnella, Nicoletta Guaragnella

**Affiliations:** 1 Department of Electrical and Information Engineering (DEI), Polytechnic of Bari, Via E. Orabona 4, 70125 Bari, Italy; ehtisham.wahid@poliba.it (E.W.); a.longo70@phd.poliba.it (A.L.); 2 Department of Biosciences, Biotechnology and Environment, University of Bari, Via E. Orabona 4, 70125 Bari, Italy; ohiemi.ocheja@uniba.it; 3 Nottingham Ningbo China Beacons of Excellence Research and Innovation Institute, University of Nottingham Ningbo China, Ningbo 315100, China; enrico.marsili@nottingham.edu.cn; 4 Institute of Physical and Chemical Processes of Italy (National Research Council—IPCF), via E. Orabona 4, 70125 Bari, Italy; massimo.trotta@cnr.it; 5 Department of Chemistry, University of Bari, Via E. Orabona 4, 70125 Bari, Italy; matteo.grattieri@uniba.it

**Keywords:** *Saccharomyces cerevisiae*, copper detection, electrochemical biosensors, cell immobilization, prototype, web-application

## Abstract

Copper contamination in the environment poses significant risks to both soil and human health, making the need for reliable monitoring methods crucial. In this study, we report the use of the EmStat Pico module as potentiostat to develop a portable electrochemical biosensor for copper detection, utilizing yeast *Saccharomyces cerevisiae* cells immobilized on a polydopamine (PDA)-coated screen-printed electrode (SPE). By optimizing the sensor design with a horizontal assembly and the volume reduction in the electrolyte solution, we achieved a 10-fold increase in current density with higher range of copper concentrations (0–300 µM CuSO_4_) compared to traditional (or previous) vertical dipping setups. Additionally, the use of genetically engineered copper-responsive yeast cells further improved sensor performance, with the recombinant strain showing a 1.7-fold increase in current density over the wild-type strain. The biosensor demonstrated excellent reproducibility (*R*^2^ > 0.95) and linearity over a broad range of copper concentrations, making it suitable for precise quantitative analysis. To further enhance portability and usability, a Bluetooth-enabled electrochemical platform was integrated with a web application for real-time data analysis, enabling on-site monitoring and providing a reliable, cost-effective tool for copper detection in real world settings. This system offers a promising solution for addressing the growing need for efficient environmental monitoring, especially in agriculture.

## 1. Introduction

Environmental pollution due to heavy metals has become an increasing concern with severe consequences for global health. Among heavy metals, copper is widely used to formulate pesticides and fungicides in farming [1]. While copper is an essential micronutrient for plant growth, its overaccumulation in soil and water can lead to toxicity, impairing plant health, reducing crop yields, and causing long-term ecological damage [2]. As such, reliable and sensitive monitoring of copper concentrations in agricultural environments is critical to ensure sustainable farming practices and to mitigate the risks of environmental and food contamination [3]. Traditional methods for copper detection, such as atomic absorption spectroscopy (AAS) and inductively coupled plasma mass spectrometry (ICP-MS), are extremely accurate but are often unsuitable for on-site or real-time monitoring due to their high costs, complex instrumentation, and the need for specialized personnel [4]. These limitations have spurred interest in the development of portable, low-cost, and user-friendly biosensors for copper quantification, as they can be used unswervingly in the field. Among the various biosensing platforms, electrochemical biosensors have gained attention for their simplicity, sensitivity, and rapid response times. These sensors typically use biological recognition elements, such as enzymes, antibodies, or living cells, to detect target analytes, producing immediate measurable electrical signals [5]. The budding yeast *Saccharomyces cerevisiae* has emerged as a promising bioreporter for electrochemical biosensors due to its well-characterized metabolic activity and ability to interact with heavy metals like copper [5,6]. One of the bottlenecks in the development of whole cell biosensors is the immobilization of the bioreporter on the transducer surface, i.e., electrodes. Particularly, the use of polydopamine (PDA) as a biocompatible coating material for immobilizing yeast cells on electrode surfaces provided an interface for analyte–biological recognition element for copper biosensors [7]. PDA mimics the natural adhesive properties of mussel foot proteins, providing a robust and stable matrix for cell attachment, while also facilitating efficient electron transfer between the cells and the electrode surface [8,9,10,11,12]. Previously, we validated the potential of PDA-immobilized *S. cerevisiae* cells for copper detection through electrochemical techniques such as cyclic voltammetry (CV) and chronoamperometry (CA), demonstrating that these sensors are capable of providing reliable, sensitive, and reproducible results [7,13]. Apart from this, recent developments in whole cell biosensing have shown that engineered *S. cerevisiae* cells, immobilized in alginate beads, can provide a visual readout of copper concentrations via colorimetric changes. These devices, which do not require electronic instrumentation, offer field-friendly deployment but often lack the sensitivity and precision needed for trace analysis [14]. Other studies have explored synthetic biology-driven modifications to the yeast copper-sensing machinery, such as incorporating copper-inducible promoters under tight transcriptional control and enabling digital, binary outputs for clear threshold-based detection [15]. Although these systems reduce background signal variability, they typically sacrifice granularity and sensitivity over a continuous range of concentrations.

A different line of research has investigated microbial fuel cell (MFC)-based biosensors that rely on electron transfer modulation by heavy metal ions to generate an electrical signal [16]. These systems are inherently low-power and can be miniaturized; however, their performance is often influenced by a complex matrix of interfering ions and microbial community dynamics, limiting their selectivity in real-world environmental samples [17]. Additionally, advanced nanomaterial-integrated electrochemical sensors, such as those employing polypyrrole–copper composites or graphene oxide-based electrodes, have demonstrated excellent sensitivity and stability for Cu (II) detection in laboratory settings [18]. The integration of chemosensors and biosensors into effect-directed analysis (EDA), in which toxicity testing is carried out prior to chemical analysis, allows screening out the biotoxic pollutants [19]. Yeast-based biosensors might be used to detect specific fungicide commonly used in modern agriculture [20]. Being a strong anhydrobiotic, *S. cerevisiae* might find specific application in environmental biosensors and under extreme desiccation conditions [21]. However, yeast-based biosensors have several challenges, among which is specificity [5]. To improve specificity, a promising approach involves next-generation eukaryotic biosensors based on engineered cells. In a previous study the authors described a whole-cell biosensor based on fluorescent detection system by engineering the promoter of *CUP1* gene and its trans-activator Cup2 in *S. cerevisiae* [22]. Yeast resists toxic concentrations of copper by activating *CUP1* gene, which encodes Cu-methalotein, a 61-amino acid protein which binds copper and cadmium [23,24]. Interestingly, the *CUP1* promoter is bound by only Cup2 and Hsf1 trans-activators, hence it responds only to toxic copper levels and heat shock. Cup2 activates *CUP1* gene in the presence of toxic levels of copper [25]. Therefore, by modifying Cup2 trans-activator, it allows to modulate the cellular mechanisms of copper detection. Nevertheless, the integration of this synthetic regulatory network into portable and field-deployable platforms remains limited [22].

In this study, we build upon the foundational work of PDA-immobilized *S. cerevisiae* cells and further develop a portable electrochemical biosensor specifically designed for copper detection. By optimizing and modifying the experimental setup, we aimed to improve the sensitivity and the electrochemical response of the biosensor, making it more suitable for field applications. In addition, we prepared genetically engineered yeast cells with enhanced copper-responsive pathways to further increase the sensitivity of the sensor. To improve the practicality of the biosensor, we integrated it with a portable, Bluetooth-enabled electrochemical platform, enabling real-time data interpretation and display through a user-friendly web application. This development facilitates on-site monitoring, providing a deployable tool for the detection of copper contamination.

## 2. Materials and Methods

### 2.1. Materials and Reagents

In this work, immobilization of cells on SPEs was achieved with dopamine hydrochloride (CAS: 62-31-7, Purity (TLC): ≥ 98%) from Sigma-Aldrich (Darmstadt, Germany). The electrochemical measurements were carried out using an electrolyte solution containing 3-(N-Morpholino) propane sulfonic acid (MOPS) buffer (pH 7), 10 mM MgCl_2_ and 50 mM glucose.

### 2.2. Electrochemical Setup

ItalSens Carbon screen-printed electrode (SPE) (Ref. IS-C-3MM) featuring 3 mm diameter carbon working electrode (WE), carbon counter electrode (CE), silver (Ag) pseudo-reference electrodes (RE), along with an EmStat Pico Development Board (Palmsens Houten, The Netherlands) were deployed for biosensor preparation and electrochemical characterization, respectively [26]. Standard sensor cable with 2 mm banana pin was used to connect electrodes attached to SPE connectors with EmStat Pico development board. EmStat Pico development board was powered by connecting it with a laptop using a USB cable and instructions were passed using Multitrace 4.5 (Palmsens, Houten, The Netherlands) software.

### 2.3. Yeast Strains and Culture Conditions

In this study, the *S. cerevisiae* W303-1B strain (MATα ade2 leu2 his3 trp1 ura3) has been used. Wild-type (WT) cells were transformed with plasmids carrying *CUP1* promoter and *CUP1* promoter with overexpression of Cup2 trans-activator to create two engineered strains, namely C1 and C2, respectively (Gietz and Schiestl (2007) [27]. The recombinant plasmids were generously donated by Valle’s lab [22].

WT strain was cultivated in either YPD (1% yeast extract, 2% bactopeptone, Gibco, Life Technologies, Waltham, MA, USA) and 2% glucose (Sigma-Aldrich, St. Louis, MO, USA) or SCD (6.70 g L^−1^ Difco Yeast nitrogen base without amino acids, 20.0 g L^−1^ glucose, 1.32 g L^−1^ Drop-out Mix without Histidine, Leucine, Lysine, Tryptophan and Uracil (USBiological life Sciences, Salem, MA, USA). Histidine, Lysine, Tryptophan, and Uracil were added to the medium at a final concentration of 76 mg L^−1^, while Leucine was added at a final concentration of 380 mg L^−1^. The recombinant strains were cultivated in SCD medium without Uracil (SCD-Ura). Each cell type was inoculated overnight and, the day after, a fresh culture with starting optical density of 0.05 was incubated at 30 °C in a shaking incubator at 180 rpm up to 7 generations, which is 14 h for WT and 18 h for C1 and C2 cells. Prior to being immobilized in PDA coatings, yeast cells underwent two centrifugation steps at 4000 g (Sorvall ST 8R, Thermo Fisher Scientific, Waltham, MA, USA) and were subsequently resuspended in electrolyte solution to eliminate extracellular debris that could influence electrochemical characterization.

### 2.4. Cell Immobilization and Electrochemical Characterization

The immobilization of cells on the surface of WE was achieved using the protocols presented in our previous work involving 1 h aerobic polymerization of dopamine followed by 20 cycles of cyclic voltammetry, which demonstrated the formation of a redox-active PDA matrix enabling stable biological immobilization and efficient electron transfer, with some modifications [7,13,28,29]. Briefly, cells grown up to the exponential phase in YPD or SCD media, with or without uracil supplementation (±Ura), were collected by centrifugation at 4000 g for 20 min. The cell pellets were resuspended in 1 mL electrolyte solution containing 20 mM MOPS buffer (pH 7), 10 mM MgCl_2_, and 50 mM glucose and then centrifuged at 10,000 g for 10 min. Cell pellet was resuspended (2 g mL^−1^) in the same electrolyte with adjusted pH to 8, as it supports the self-polymerization of PDA. This cell suspension was then mixed in a 1:1 ratio with a 10 mM solution of dopamine hydrochloride made in the same electrolyte (pH 8) and stirred magnetically under aerobic conditions for one hour for aerobic polymerization. After the aerobic polymerization, 5 μL aliquot of the mixture was applied onto the WE surface of a SPE and left to dry at 26 ± 1 °C for 60 min to obtain a bio-coating. Electrochemical polymerization was then conducted using 20 cycles of CV from −0.3 V to +0.5 V at a scan rate of 20 mV s^−1^, followed by electrochemical characterization using CA at 0.4 V. Differently from our previous protocols, most of the experiments in current studies have been carried out by positioning the biosensor horizontally: the electrolyte solution (100 μL) with varying copper concentrations was placed directly onto the sensor surface of the bio-coated electrode. All procedures were carried out at a controlled temperature of 26 ± 1 °C and each experiment was conducted at least in triplicate. Cell cultures were separately grown under the identical conditions to obtain independent biological replicates. All electrochemical tests were performed making use of freshly prepared SPEs. While detailed physicochemical characterization of the PDA coatings (e.g., via Scanning Electron Microscopy (SEM) or X-ray photoelectron spectroscopy (XPS) was not performed in this study, a similar PDA-based yeast immobilization approach was characterized by SEM in our previous work to confirm uniform cell coverage and coating morphology [7].

### 2.5. Data Analysis

For each dataset, the mean and standard deviation were calculated using OriginPro, version 8.5.1 (OriginLab Corporation, Northampton, MA, USA) from three independent biological replicates (n = 3). The data was analyzed for linear fitting at 1000 s and 2500 s, based on experimental assembly (i.e., horizontal and vertical, respectively) to obtain *R*^2^ value, and LoD (Limit of Detection) values were calculated according to the 3*S_b_/m* criterion, where *m* is the slope of the linear range and *S_b_* the standard deviation of the intercept [7]. For prototype, the data was analyzed with web application developed using Python, version 3.10 (Python Software Foundation, Wilmington, DE, USA) [28].

## 3. Results and Discussion

### 3.1. Development of EmStat Pico System for Chronoamperometric Analysis of Yeast S. cerevisiae Cells Immobilized on a PDA-Coated SPE

**Figure 1:**
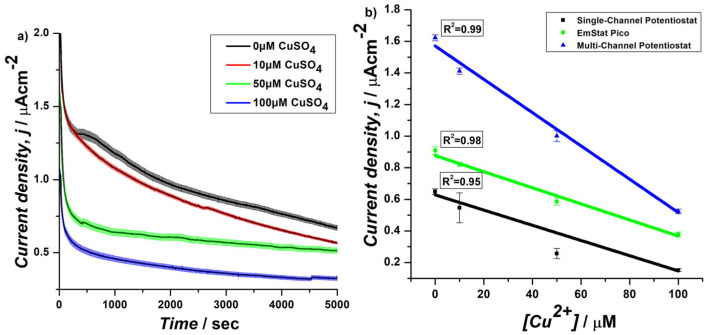
CA at 0.4 V of *S. cerevisiae* yeast cells cultured in YPD and immobilized on a PDA-coated SPE. (**a**) Current density measurements were performed by using the EmStat Pico module as potentiostat in the presence of different concentrations of CuSO_4_. (**b**) Relationship between current density and CuSO_4_ concentrations and comparison of correlation coefficients (*R*^2^) for single-channel, EmStat Pico, and multi-channel potentiostats during chronoamperometry at 2500 s.

With the aim to develop portable PoC electrochemical devices, the biosensors were prepared by immobilizing yeast cells using PDA, which in our previous study and in the literature has shown effective immobilization by film morphological uniformity and surface chemical composition [7,30]. The characterization of biosensors was performed using the EmStat Pico development board [26]. The first set of experiments focused on the CA (Figure 1a) of biosensors prepared with WT cells cultured in YPD placed vertically in 18 mL electrolyte solution (containing MOPS buffer (pH 7), 10 mM MgCl_2_, and 50 mM glucose) with different concentrations of copper (0–100 uM) and the results were compared to previous studies using single- and multi-channel potentiostats (Figure 1b) [7,13]. The principle of detection is based on the metabolic activity of immobilized *S. cerevisiae* cells and the inhibitory effect of heavy metals, such as copper ions, on this activity, which can be quantified electrochemically. At an applied potential of +0.4 V vs. Ag pseudo-reference electrode, the *S. cerevisiae* cells metabolize available carbon sources (e.g., glucose) through their native respiratory pathways, generating intracellular electrons. Part of this electron flow is transferred extracellularly to the electrode via the PDA coating, which serves as a conductive and biocompatible interface. This configuration enables direct electron transfer (DET) from yeast to electrode, without the use of exogenous redox mediators. Cu^2+^ ions interfere with the cellular respiration of yeast, particularly at the mitochondrial level, resulting in a reduced electron flow and therefore a decrease in current response. This inhibition-based signal is used to quantify copper concentration [7,31].

The data was analyzed for linear fitting at 2500 s to obtain *R*^2^ value and LoD values were calculated (Tables S1 and S2). Results showed a 1.7-fold decrease (Table S3) in current density and 4.86-fold higher (Table S4) LoD (10.7 µM CuSO_4_) with respect to multi-channel potentiostat system (2.2 µM CuSO_4_). Despite lower current densities and higher LoD, the *R*^2^ values obtained from both systems were similar (Figure 1b). Specifically, the lower current density and higher LoD value is attributed to the absence of redox mediator, which has been used in multi-channel potentiostat systems [7]. In fact, when the EmStat Pico system was compared with a single-channel potentiostat system, it presented a 1.4-fold increase in current density (Table S3) and 1.6-fold increase in LoD (Table S4) with an improved *R*^2^ value. The improvement in current density and lowering of LoD value is due to both the use of SPE and of a miniaturized system that can provide lower resistance for EET [26,32]. The higher *R*^2^ with respect to single-channel system and consistency with respect to multi-channel system indicates the reproducibility and robustness of the EmStat Pico board platform in providing a reliable correlation between the concentration of Cu (II) and the electrochemical signal, similar to previous findings. It is also worth noting that the EmStat Pico system offers a significant advantage in terms of portability, which is crucial for field applications. However, it was observed that the traditional electrode vertical setup requires high volumes of electrolyte and only provides quantification between 0 and 100 uM copper concentrations and with low sensitivity.

**Figure 2:**
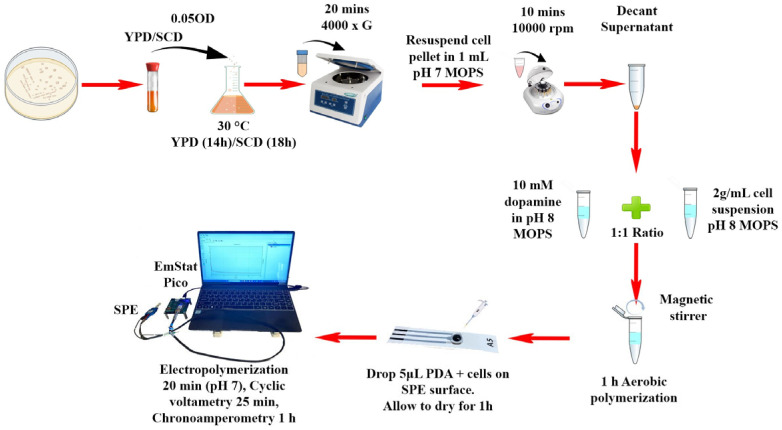
Modified protocol for immobilization of *S. cerevisiae* yeast cells on SPE using PDA matrix and schematic representation of horizontal assembly for electrochemical characterization of biosensors using EmStat Pico development board.

**Figure 3:**
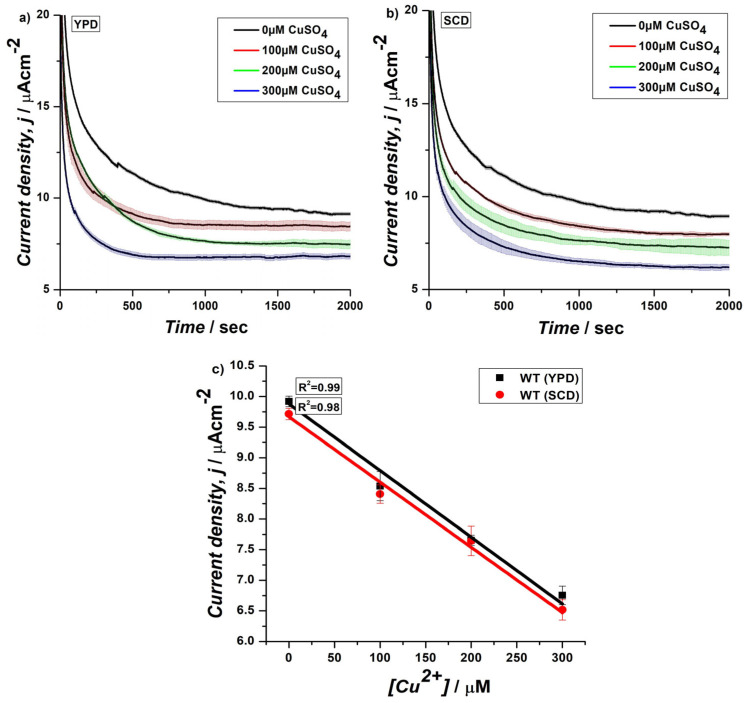
CA at 0.4 V of *S. cerevisiae* yeast cells cultured (**a**) in YPD or (**b**) in SCD, and immobilized on a PDA-coated SPE in the presence of different concentrations of CuSO_4_ under aerobic conditions using EmStat Pico system with horizontal assembly, (**c**) relationship between current density and CuSO_4_ concentrations and comparison of correlation coefficients (*R*^2^) for cell growth in YPD or SCD at 1000 s.

With the goal to improve the range of detection and sensitivity, we explored an alternative assembly for CA of the biosensor. The cells, cultured in YPD or SCD medium, were normally immobilized onto the electrode surface by using PDA, but unlike the traditional vertical dipping method, the experimental setup was modified by placing the biosensor horizontally, and 100 µL of electrolyte with increasing concentrations of copper (0, 100, 200, 300 µM) were placed onto its surface (Figure 2). The CA was performed for 2000 s and the data were analyzed at 1000 s for linear fitting and calculation of LoD values (Figure 3 and Tables S5–S8). These aimed to evaluate the performance of the biosensor while minimizing the volume of electrolyte, time of analysis, and by improving copper range, which are particularly relevant for field applications where volumes, time, and range are important. Biosensors were prepared by immobilizing WT cells cultured in YPD or SCD media and LoD values of 25.6 and 25.7 uM CuSO_4_ and *R*^2^ values of 0.99 and 0.98 over a range from 0 to 300 uM CuSO_4_ were obtained, respectively (Figure 3c and Tables S5–S8). The results from this modified setup demonstrated a striking improvement of approximately 10-fold in current densities at 0 and 100 µM (Tables S9 and S10) in both cases, when compared to the previous result, which utilized a similar PDA-immobilized yeast cell configuration but with the traditional vertical dipping approach.

Interestingly, the results obtained from the biosensors cultured in SCD medium showed no significant difference in performance when compared to those prepared using YPD medium ((Figure 3c) and Tables S5 and S8), indicating that both media provide sufficient nutrients for yeast cell growth and metabolic activity [33] and do not affect the electrochemical response. This experiment was also preliminary to the next test on genetically engineered yeast cells, for which a selective medium has to be used. Overall, improvement in current density observed with the modified setup can be attributed to several factors. First, the horizontal placement of the sensor, along with localized application of 100 µL of electrolyte directly onto the biosensor surface, possibly optimized the contact between the biosensor and the electrolyte. This targeted application may have enhanced the diffusion of copper ions to the electrode surface, facilitating more efficient electron transfer, reducing resistance, and allowing for a stronger signal [34]. Furthermore, the horizontal arrangement of the biosensor could have allowed for better utilization of the immobilized yeast cells’ catalytic activity, thereby enhancing the overall current response [35]. Despite the substantial improvement in current density, the *R*^2^ value remained consistent with previous observations, indicating that the modified experimental setup did not compromise the reproducibility or linearity of the biosensor’s response to Cu (II) ions, similar to the vertical dipping method. Moreover, the significant improvement in current density, without compromising the *R*^2^ value, represented a meaningful advancement in the development of EmStat Pico-based prototype setup. This configuration also aligns with the goal of enhancing the portability and practical usability of the sensor, as it offers a more controlled and efficient electrochemical measurement method.

### 3.2. CA of Genetically Engineered Yeast Cells on SPEs Using EmStat PicoSsystem in Horizontal Assembly

**Figure 4:**
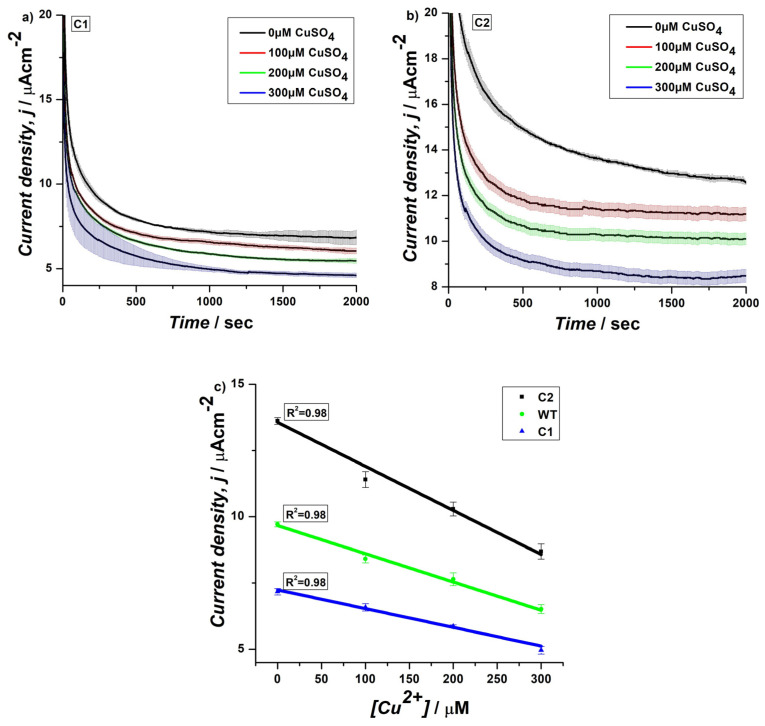
CA at 0.4 V of *S. cerevisiae* engineered yeast cells (**a**) C1 and (**b**) C2 cultured in SCD-Ura with different concentrations of CuSO_4_ under aerobic conditions using EmStat Pico system with horizontal assembly, (**c**) relationship between current density and CuSO_4_ concentrations and comparison of correlation coefficients (*R*^2^) for C1, C2, and WT cells at 1000 s.

In order to further optimize the sensitivity of the yeast electrochemical biosensor for copper monitoring, we prepared genetically engineered strains over-expressing CUP1 promoter (C1) and both CUP1 promoter and its trans-activator Cup2 (C2). CUP1 gene expression is induced in the presence of a copper excess, and its activation is mediated by the transcription factor Cup2, which is essential for copper resistance [36]. Engineered strains were grown in selective SCD medium and regularly immobilized onto the electrode surface with PDA. The electrochemical measurements by CA were performed with the horizontal assembly of the system (Figure 4a,b) for 2000 s, and the data obtained were analyzed at 1000 s for linear fitting and calculation of LoD values, as in previous experiments (Tables S11–S14). Results revealed a notable difference in performance between biosensors prepared using recombinant strains and WT cells (Table S15). The biosensors prepared using C1 (LoD = 43, *R*^2^ = 0.98) showed the lowest current density with a minimum of a 0.73-fold decrease in current density and 1.7-fold lower LoD compared to WT cells (Table S15). On the other hand, the copper-specific strains C2 (LoD = 26.8, *R*^2^ = 0.98) exhibited a 1.7-fold increase in current density (Table S15) and 1.6-fold lower LoD compared to the C1 (Figure 4c). This significant increase in current density suggests that the C2 strain, having the Cup2 trans-activator for enhanced copper responsiveness, is more efficient in catalyzing the electrochemical reaction in the presence of Cu(II) ions. Results obtained on the recombinant strains indicate that CUP1 over-expression itself has a detrimental effect on the electrochemical performance of the biosensor, while in the presence of Cup2, a significant improvement of the electrochemical parameters, in terms of current density and copper sensitivity, could be detected. The higher current density in C2 cells indicates a more effective capacity to transfer electrons during the redox process, likely due to the activation of copper-specific enzymes and pathways that enhance the strain’s interaction with Cu(II) ions [22]. Furthermore, the C2 strain also showed a 1.3-fold increase (Table S15) in current density than the WT strain, with similar LoD and *R*^2^ values (Figure 4c and Tables S8 and S14). This improvement indicates that the genetic modifications made to the C2 strain contributed to an enhanced electrochemical response when compared to the WT cells. The similarity in *R*^2^ values between C2 and WT suggests that the genetic modifications did not negatively impact the reproducibility or linearity of the biosensor’s response. Instead, the improvements in current density highlight the potential of using genetically engineered strains for more sensitive and efficient biosensing applications, particularly for detecting copper ions in environmental or industrial samples. The consistent *R*^2^ values across all strains also emphasize the reliability and robustness of the electrochemical measurements, ensuring that the biosensor can provide precise quantitative analysis, regardless of the strain used. This is crucial for both laboratory research and potential field applications where accuracy and reproducibility are essential. It is worth mentioning here that the sensors developed in this study exhibit moderate LoD (≥2.2 µM). However, they demonstrate improved sensitivity compared to certain commercially available test kits, such as the MQuant^®^ dipsticks (LoD ≥≥ 157.4). While we do not achieve the ultra-high sensitivity levels reported for some nanostructured or enzymatic systems, the sensors present distinct advantages in terms of long-term applicability, portability, and the potential to assess copper in agricultural water samples (Table S16).

### 3.3. Transferability of Yeast-Based Biosensors System to Portable Microelectronic Device

**Figure 5:**
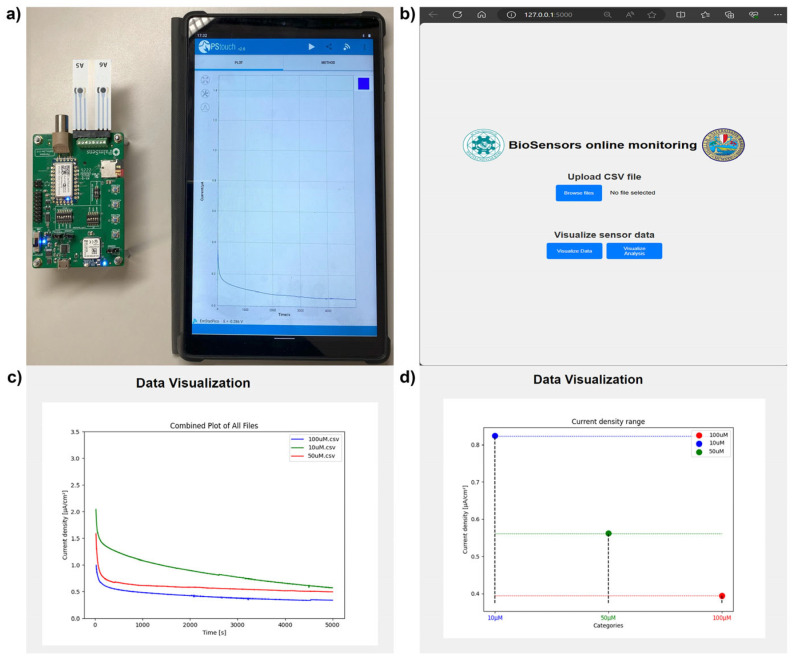
(**a**) Working prototype of handheld device with in-house biosensors connected to Android tablet for electrochemical quantification of CuSO_4_, (**b**) display of index page of web application, (**c**) display of data visualization page of web application for i-T trace, (**d**) display of data visualization page of web application for classification of sample into different categories based on current density values at 2500 s of CA data [28].

Results obtained from the electrochemical characterization of biosensors prepared by using different cell types, such as WT and recombinant strains, paved the way for the development of form factor prototype for PoC application for detection of different concentrations of copper. To this aim, SPE connectors were used for in-house integration of yeast-based biosensors to EmStat Pico development board, powered by 2x AAA batteries and connected to an Android tablet via Bluetooth to pass instructions using PStouch software, version 2.8 (Figure 5a). To perform real-time, online monitoring of the different concentrations of copper gathered by the proposed handheld device, a Python-based web application is also introduced (Figure 5b). The web application is platform independent and allows data to be accessed and visualized by any device having a web browser (Figure 5c,d).

**Figure 6:**
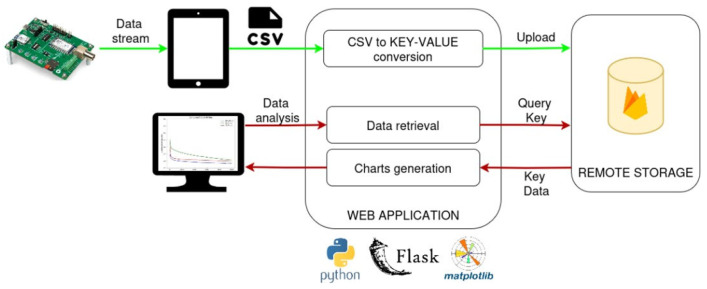
Working information flow and system architecture of the proposed Python-based web application for real-time monitoring of different concentrations of copper captured by the developed handheld device [28].

The system’s backend logic leverages Python Flask, an open-source micro-framework for building web applications using the Python language, while the data layer relies on Google Firebase for remote data storage and management [37]. Firebase runs on Google’s infrastructure and its cloud to provide a suite of tools to develop and manage cross-platform applications. The database interacts with the Python flask application that handles all the CRUD (create, read, update, and delete) operations. Moreover, its key-value NoSQL paradigm makes it suitable to seamlessly interact with Python data structures. Figure 6 illustrates the information flow within the proposed web application, starting from the data collection system. This process, running on board the EmStat Pico board, generates a .csv file containing all the sensor readings. Once the file is ready, the web application User Interface (UI) allows browsing of the filesystem and selection of the .csv file to be uploaded to the remote database. This routine first converts the input file into a format that is suitable for the Firebase NoSQL: a new key having the name of the .csv file is created in the database root, and all the current density data is serialized and stored as value associated with the key. Once all data is uploaded, it can therefore be retrieved via file name.

Besides data uploading, the web application also offers Data Visualization and Data Analysis functionalities, allowing users to view current density vs. time curves, and analyze concentration levels of copper based on CA data (Figure 5b). When the user asks to visualize data, a routine based on JavaScript engine fetches all the stored filenames from the database and prompts a list of checkboxes where the user can select only the files to visualize and compare. When the subset of data to be visualized is selected, the user is redirected to the data visualization page. Here current density curves are plotted over time, and a box plot is generated to show the distribution of current density values at a specific time point (2500 s), allowing users to classify samples into different concentration categories of CuSO_4_ (Figure 5c,d). To do so, the Python backend logic inputs all file keys and retrieves from the real-time database all the values associated with each. The matplotlib python library is then used to create visualization charts. At this stage, if the user request is to visualize the current density curves over time, all current density values are stored in a list, and a cumulative plot is created by overlapping the current density curves from all the selected files. If the user request is to visualize current density distribution, only the current density values corresponding to time, for instance 2500 s, are collected for each file and displayed as a scatterplot. To render the charts on the webpage UI, the base64 Python library is utilized, as it bypasses saving the matplotlib-generated image by using the Input/Output buffer directly [28].

The testing of the device along with web application was performed using the known concentration of copper. The CA was carried out at 0.4 V for a total of 5000 s using biosensors prepared by immobilizing WT cells on SPEs integrated to EmStat Pico development board, measuring current density changes as the concentration of CuSO_4_ varied [28]. The obtained data was saved in .csv format in the tablet and analyzed using web application. The application was able to distinguish between different known concentrations of copper and display them in the form of a line graph and a box plot (Figure 5d).

## 4. Conclusions

This work demonstrates the successful development of a portable electrochemical biosensor for the detection of copper in environmental samples, addressing the need for efficient and real-time monitoring tools. By immobilizing *S. cerevisiae* yeast cells on a PDA-coated carbon SPE, we optimized the sensor performance by testing a horizontal assembly and reducing the volume of electrolyte. This resulted in a notable improvement in both current density and sensitivity. The application of genetically engineered yeast strains further enhanced the biosensor’s sensitivity to copper ions. The biosensor displayed high reproducibility and linearity over a wider range of copper concentrations, making it suitable for precise and reliable detection. Additionally, the incorporation of a Bluetooth-enabled platform and a web application for data analysis facilitates on-site monitoring, offering a user-friendly and cost-effective solution for in-field applications, especially in resource-limited settings. It is worth mentioning here that the present study was conducted in a controlled MOPS buffer system to evaluate the fundamental electrochemical response of the yeast-based biosensor to Cu^2+^. The coexisting ions (e.g., Zn^2+^, Pb^2+^, Ca^2+^, etc.) commonly found in environmental matrices may affect selectivity and could potentially induce cross-reactivity. The investigations along with spike testing, validation of sensor performance under field-relevant environmental conditions, and conducting long-term monitoring will be critical in translating the current platform to field applications and will be reported in a future study. However, this system represents a promising advancement in the development of portable biosensors, with the potential to provide an accessible tool and contribute to sustainable practices for managing copper toxicity in different areas of application from environmental, agricultural, and clinical diagnostics.

## Data Availability

The data are available upon request.

## Supplementary Materials

The following supporting information can be downloaded at: https://www.mdpi.com/article/10.3390/bios15090583/s1, Table S1: Current densities at 2500 s for biosensors prepared using WT S. cerevisiae cells cultured in YPD characterised using EmStat Pico in vertical assembly; Table S2: Linear fitting and LoD calculations for current densities obtained at 2500 s for biosensors prepared using WT cells cultured in YPD and characterised using EmStat Pico in vertical assembly; Table S3: Comparison of current densities at 2500 s for biosensors prepared using WT cells cultured in YPD and characterised using EmStat Pico, single channel and multichannel potentiostat in vertical assembly; Table S4: Comparison of LoD for biosensors prepared using WT cells cultured in YPD and characterised using sin-gle channel and multichannel potentiostat with EmStat Pico; Table S5: Current densities at 1000 s for biosensors prepared using WT cells cultured in YPD and characterised us-ing EmStat Pico in horizontal assembly; Table S6: Linear fitting and LoD calculations for current densities obtained at 1000 s for biosensors prepared using WT cells cultured in YPD and characterised using EmStat Pico in horizontal assembly; Table S7: Current Densities at 1000 s for biosensors prepared using WT cells cultured in SCD and characterised us-ing EmStat Pico in horizontal assembly; Table S8: Linear fitting and LoD calculations for current densities obtained at 1000 s for biosensors prepared using WT cells cultured in SCD and characterised using EmStat Pico in horizontal assembly; Table S9: Comparison of current densities for biosensors prepared using WT cells cultured in YPD and characterised using EmStat Pico in horizontal and vertical assemblies; Table S10: Comparison of current densities for biosensors prepared using WT cells cultured in SCD and YPD and characterised using EmStat Pico in horizontal and vertical assemblies, respectively; Table S11: Current Densities at 1000 s for biosensors prepared using C1 cells cultured in SCD-Ura and character-ised using EmStat Pico in horizontal assembly; Table S12: Linear fitting and LoD calculations for current densities obtained at 1000 s for biosensors prepared using C1 cells cultured in SCD-Ura and characterised using EmStat Pico in horizontal assembly; Table S13: Current Densities at 1000 s for biosensors prepared using C2 cells cultured in SCD-Ura and character-ised using EmStat Pico in horizontal assembly; Table S14: Linear fitting and LoD calculations for current densities obtained at 1000 s for biosensors prepared us-ing C2 cells cultured in SCD-Ura and characterised using EmStat Pico in horizontal assembly; Table S15: Comparison of current densities for biosensors prepared using C1, C2 and WT cells characterised using EmStat Pico in horizontal assemblies; Table S16: Comparison Table: Enzyme/Nanomaterial-based sensors and commercially available copper detection methods (2020–2025). References [38,39,40,41,42,43,44,45,46] are cited in Supplementary Materials.

## References

[1] Alengebawy A., Abdelkhalek S.T., Qureshi S.R., Wang M.-Q. (2021). Heavy Metals and Pesticides Toxicity in Agricultural Soil and Plants: Ecological Risks and Human Health Implications. Toxics.

[2] Rehman M., Liu L., Wang Q., Saleem M.H., Bashir S., Ullah S., Peng D. (2019). Copper environmental toxicology, recent advances, and future outlook: A review. Environ. Sci. Pollut. Res..

[3] Rippa M., Battaglia V., Cermola M., Sicignano M., Lahoz E., Mormile P. (2022). Monitoring of the copper persistence on plant leaves using pulsed thermography. Environ. Monit. Assess..

[4] Alharbi A., Sari A.A., Alessa A.H., Snari R.M., Alsharief H.H., Alatawi I.S., El-Zaidia E.F.M., El-Metwaly N.M. (2024). High-sensitivity detection of copper ions in water via cellulose nanomaterial nano-antennas and DFT studies. Chem. Eng. J. Adv..

[5] Wahid E., Ocheja O.B., Marsili E., Guaragnella C., Guaragnella N. (2022). Biological and technical challenges for implementation of yeast-based biosensors. Microb. Biotechnol..

[6] Jarque S., Bittner M., Blaha L., Hilscherova K. (2016). Yeast Biosensors for Detection of Environmental Pollutants: Current State and Limitations. Trends Biotechnol..

[7] Wahid E., Ocheja O.B., Oguntomi S.O., Pan R., Grattieri M., Guaragnella N., Guaragnella C., Marsili E. (2025). Immobilized Saccharomyces cerevisiae viable cells for electrochemical biosensing of Cu(II). Sci. Rep..

[8] Holten-Andersen N., Waite J.H. (2008). Mussel-designed Protective Coatings for Compliant Substrates. J. Dent. Res..

[9] Papov V.V., Diamond T.V., Biemann K., Waite J.H. (1995). Hydroxyarginine-containing Polyphenolic Proteins in the Adhesive Plaques of the Marine Mussel Mytilus edulis. J. Biol. Chem..

[10] Liu Y., Ai K., Lu L. (2014). Polydopamine and Its Derivative Materials: Synthesis and Promising Applications in Energy, Environmental, and Biomedical Fields. Chem. Rev..

[11] Yang S.H., Kang S.M., Lee K.-B., Chung T.D., Lee H., Choi I.S. (2011). Mussel-Inspired Encapsulation and Functionalization of Individual Yeast Cells. J. Am. Chem. Soc..

[12] Fakhrullin R.F., Zamaleeva A.I., Minullina R.T., Konnova S.A., Paunov V.N. (2012). Cyborg cells: Functionalisation of living cells with polymers and nanomaterials. Chem. Soc. Rev..

[13] Ocheja O.B., Wahid E., Franco J.H., Trotta M., Guaragnella C., Marsili E., Guaragnella N., Grattieri M. (2024). Polydopamine-immobilized yeast cells for portable electrochemical biosensors applied in environmental copper sensing. Bioelectrochemistry.

[14] Vopálenská I., Váchová L., Palková Z. (2015). New biosensor for detection of copper ions in water based on immobilized genetically modified yeast cells. Biosens. Bioelectron..

[15] Fan C., Zhang D., Mo Q., Yuan J. (2022). Engineering *Saccharomyces cerevisiae*-based biosensors for copper detection. Microb. Biotechnol..

[16] Franco J.H., Stufano P., Labarile R., Lacalamita D., Lasala P., Fanizza E., Trotta M., Farinola G.M., Grattieri M. (2024). Intact photosynthetic bacteria-based electrodes for self-powered metal ions monitoring. Biosens. Bioelectron. X.

[17] Adekunle A., Raghavan V., Tartakovsky B. (2019). On-line monitoring of heavy metals-related toxicity with a microbial fuel cell biosensor. Biosens. Bioelectron..

[18] Timoshenko R.V., Gorelkin P.V., Vaneev A.N., Krasnovskaya O.O., Akasov R.A., Garanina A.S., Khochenkov D.A., Iakimova T.M., Klyachko N.L., Abakumova T.O. (2023). Electrochemical Nanopipette Sensor for In Vitro/In Vivo Detection of Cu^2+^ Ions. Anal. Chem..

[19] Li M., Guo L.H. (2023). Chemo/biosensors towards effect-directed analysis: An overview of current status and future development. TrAC Trends Anal. Chemistry.

[20] Mendes F., Miranda E., Amaral L., Carvalho C., Castro B.B., Sousa M.J., Chaves S.R. (2024). Novel yeast-based biosensor for environmental monitoring of tebuconazole. Appl. Microbiol. Biotechnol..

[21] Maria S.R.S., Marina D.B., Tieze S.M., Liddell L.C., Bhattacharya S. (2023). BioSentinel: Long-Term *Saccharomyces cerevisiae* Preservation for a Deep Space Biosensor Mission. Astrobiology.

[22] Žunar B., Mosrin C., Bénédetti H., Vallée B. (2022). Re-engineering of CUP1 promoter and Cup2/Ace1 transactivator to convert Saccharomyces cerevisiae into a whole-cell eukaryotic biosensor capable of detecting 10 nM of bioavailable copper. Biosens. Bioelectron..

[23] Ecker D.J., Butt T.R., Sternberg E.J., Neeper M.P., Debouck C.A., Gorman J., Crooke S.T. (1986). Yeast metallothionein function in metal ion detoxification. J. Biol. Chem..

[24] Shen C.-H., Leblanc B.P., Alfieri J.A., Clark D.J. (2001). Remodeling of Yeast *CUP1* Chromatin Involves Activator-Dependent Repositioning of Nucleosomes over the Entire Gene and Flanking Sequences. Mol. Cell. Biol..

[25] Singh S., Sahu R.K., Tomar R.S. (2021). The N-Terminal Tail of Histone H^3^ Regulates Copper Homeostasis in *Saccharomyces cerevisiae*. Mol. Cell. Biol..

[26] Stratmann L., Heery B., Coffey B. EmStat Pico: Embedded Electrochemistry with a Miniaturized Software-Enabled Potentiostat System on Module. https://www.analog.com/en/resources/technical-articles/emstat-pico-embedded-electrochemistry-with-a-miniaturized-software-enabled-potentiostat-system-on-mo.html?gated=1755509085133.

[27] Gietz R.D., Schiestl R.H. (2007). High-efficiency yeast transformation using the LiAc/SS carrier DNA/PEG method. Nat. Protoc..

[28] Wahid E. (2024). Design and Characterization of Yeast-based Electrochemical Biosensors for Application in Precision Agriculture. Ph.D. Thesis.

[29] Buscemi G., Vona D., Stufano P., Labarile R., Cosma P., Agostiano A., Trotta M., Farinola G.M., Grattieri M. (2022). Bio-Inspired Redox-Adhesive Polydopamine Matrix for Intact Bacteria Biohybrid Photoanodes. ACS Appl. Mater. Interfaces.

[30] Rella S., Mazzotta E., Caroli A., De Luca M., Bucci C., Malitesta C. (2018). Investigation of polydopamine coatings by X-ray Photoelectron Spectroscopy as an effective tool for improving biomolecule conjugation. Appl. Surf. Sci..

[31] Shi H., Jiang Y., Yang Y., Peng Y., Li C. (2020). Copper metabolism in Saccharomyces cerevisiae: An update. BioMetals.

[32] Crapnell R.D., Ferrari A.G.M., Dempsey N.C., Banks C.E. (2022). Electroanalytical overview: Screen-printed electrochemical sensing platforms for the detection of vital cardiac, cancer and inflammatory biomarkers. Sens. Diagn..

[33] Eliodório K.P., Cunha G.C., Lino F.S., Sommer M.O.A., Gombert A.K., Giudici R., Basso T.O. (2023). Physiology of Saccharomyces cerevisiae during growth on industrial sugar cane molasses can be reproduced in a tailor-made defined synthetic medium. Sci. Rep..

[34] Sedlák P., Kuberský P. (2020). The Effect of the Orientation Towards Analyte Flow on Electrochemical Sensor Performance and Current Fluctuations. Sensors.

[35] Liu X., Wang K., Liu Y., Zhao F., He J., Wu H., Wu J., Liang H., Huang C. (2023). Constructing an ion-oriented channel on a zinc electrode through surface engineering. Carbon Energy.

[36] Welch J., Fogel S., Buchman C., Karin M. (1989). The CUP2 gene product regulates the expression of the CUP1 gene, coding for yeast metallothionein. EMBO J..

[37] Anshori I., Harimurti S., Rama M.B., Langelo R.E., Jessika, Yulianti L.P., Gumilar G., Yusuf M., Prastriyanti S., Yuliarto B. (2022). Web-based surface plasmon resonance signal processing system for fast analyte analysis. SoftwareX.

[38] Fu Y., Li J., Wang J., Wang E., Fang X. (2024). Development of a two component system based biosensor with high sensitivity for the detection of copper ions. Commun. Biol..

[39] Yin B., Wan X., Qian C., Sohan A.S.M.M.F., Zhou T., Yue W. (2021). Enzyme Method-Based Microfluidic Chip for the Rapid Detection of Copper Ions. Micromachines.

[40] Lucci T.J., Neufarth A., Gaillard J.-F., Lucks J.B. (2024). A Sensor for Detecting Aqueous Cu^2+^ That Functions in a Just-Add-Water Format. ACS Omega.

[41] Bendicho C., Lavilla I., Pena-Pereira F., de la Calle I., Romero V. (2021). Nanomaterial-Integrated Cellulose Platforms for Optical Sensing of Trace Metals and Anionic Species in the Environment. Sensors.

[42] Nidhisha, Kizhakayil R.N. (2025). Onsite naked-eye detection and quantification of Cu(ii) ions in drinking water using N-doped carbon nanodots. Mater. Adv..

[43] LaMotte Europe Insta Test Copper and Iron Test Strip. https://www.lamotte-europe.com/products/pool-and-spa/test-strips/insta-test-copper-and-iron-test-strip-kit/en.

[44] FStest Heavy Metal Copper(Cu) Test Strip for Water. https://www.fstestcorp.com/products-detail/id-118.html.

[45] EZ Series Copper Analysers EZ1010 Copper. https://my.hach.com/ez-series-analysers/ez-series-copper-analysers/family?productCategoryId=59429629671&utm.

[46] MeRcK Copper Test. https://www.sigmaaldrich.com/IT/en/product/mm/110003?utm=.

