# Supplementary material for: A Bluetooth-Enabled Electrochemical Platform Based on Saccharomyces cerevisiae Yeast Cells for Copper Detection
Source: Biosensors (Basel). 2025 Sep 5;15(9):583. doi: 10.3390/bios15090583 (PMC12467965; doi:10.3390/bios15090583)
Supplement: Supplementary file 1 [file biosensors-15-00583-s001.zip › biosensors-3805384-supplementary.pdf]

# A Bluetooth-Enabled Electrochemical Platform Based on *Saccharomyces cerevisiae* Yeast Cells for Copper Detection

**Ehtisham Wahid [1], Ohiemi Benjamin Ocheja [2], Antonello Longo [1], Enrico Marsili [3], Massimo Trotta [4], Matteo Grattieri [5] and Cataldo Guaragnella [1,*] and Nicoletta Guaragnella [2,*]**

1 Department of Electrical and Information Engineering (DEI), Polytechnic of Bari, Via E. Orabona 4, 70125 Bari, Italy; ehtisham.wahid@poliba.it (E.W.); a.longo70@phd.poliba.it (A.L.)
2 Department of Biosciences, Biotechnology and Environment, University of Bari, Via E. Orabona 4, 70125 Bari, Italy; ohiemi.ocheja@uniba.it
3 Nottingham Ningbo China Beacons of Excellence Research and Innovation Institute, University of Nottingham Ningbo China, Ningbo 315100, China; enrico.marsili@nottingham.edu.cn
4 Institute of Physical and Chemical Processes of Italy (National Research Council—IPCF), via E. Orabona 4, 70125 Bari, Italy; massimo.trotta@cnr.it
5 Department of Chemistry, University of Bari, Via E. Orabona 4, 70125 Bari, Italy; matteo.grattieri@uniba.it
* Correspondence: cataldo.guaragnella@poliba.it (C.G.); nicoletta.guaragnella@uniba.it (N.G.)

## Supplementary Materials

Table S1: Current densities at 2500 s for biosensors prepared using WT *S. cerevisiae* cells cultured in YPD characterised using EmStat Pico in vertical assembly.

| [Cu2+] / uM | Current Densities (WT (YPD) (EmStat)) | SD |
|---|---|---|
| **0** | 0.90874 | 0.02342 |
| **10** | 0.82172 | 0.01259 |
| **50** | 0.58664 | 0.02272 |
| **100** | 0.37728 | 0.01594 |

Table S2: Linear fitting and LoD calculations for current densities obtained at 2500 s for biosensors prepared using WT cells cultured in YPD and characterised using EmStat Pico in vertical assembly.

| **Fitting results** | | | |
|---|---|---|---|
| **Equation** | y = a + b*x | | |
| **Weight** | Instrumental | | |
| **Residual Sum of Squares** | 4.7867 | | |
| **Pearson's r** | -0.996 | | |
| **Adj. R-Square** | 0.98802 | | |
| | | Value | Standard Error |
| **B** | Intercept | 0.8768 | 0.01827 |
| **B** | Slope | -0.00509 | 3.23E-04 |
| **LoD** | -10.76817289 | | |

Table S3: Comparison of current densities at 2500 s for biosensors prepared using WT cells cultured in YPD and characterised using EmStat Pico, single channel and multichannel potentiostat in vertical assembly [7, 13].

| [Cu2+] / uM | Current Densities (WT (YPD) (EmStat)) | SD | Current Densities (WT (YPD) (single channel)) | SD | Current Densities (WT (YPD) (Multichannel)) | SD | Fold increase in EmStat w.r.t Single channel | Fold decrease in EmStat w.r.t Multichannel |
|---|---|---|---|---|---|---|---|---|
| **0** | 0.90874 | 0.02342 | 0.64964 | 0.01797 | 1.6229 | 0.0054 | 1.398836279 | 1.785879349 |
| **10** | 0.82172 | 0.01259 | | | 1.4115 | 0.0281 | | 1.717738402 |
| **20** | | | 0.50649 | 0.09484 | | | | |
| **50** | 0.58664 | 0.02272 | 0.25671 | 0.03231 | 1.0006 | 0.031 | 2.285224572 | 1.705645711 |
| **100** | 0.37728 | 0.01594 | 0.15186 | 0.00855 | 0.5235 | 0.0091 | 2.48439352 | 1.387563613 |

Table S4: Comparison of LoD for biosensors prepared using WT cells cultured in YPD and characterised using single channel and multichannel potentiostat with EmStat Pico [7, 13].

| System | LoD | Comparison | Fold change in LOD | |
|---|---|---|---|---|
| **Multichannel system** | 2.2 | **EmStat vs Multichannel** | 4.863636364 | Decrease |
| **EmStat** | 10.7 | | | |
| **Single channel system** | 12.5 | **EmStat vs single channel** | 1.168224299 | Increase |

Table S5: Current densities at 1000 s for biosensors prepared using WT cells cultured in YPD and characterised using EmStat Pico in horizontal assembly.

| [Cu2+] / uM | WT(YPD) Horizontal | SD |
|---|---|---|
| **0** | 9.91906 | 0.08635 |
| **100** | 8.53799 | 0.23961 |
| **200** | 7.65254 | 0.08472 |
| **300** | 6.75335 | 0.15035 |

Table S6: Linear fitting and LoD calculations for current densities obtained at 1000 s for biosensors prepared using WT cells cultured in YPD and characterised using EmStat Pico in horizontal assembly.

| Fitting results WT-YPD | | | |
|---|---|---|---|
| **Equation** | y = a + b*x | | |
| **Weight** | Instrumental | | |
| **Residual Sum of Squares** | 2.53171 | | |
| **Pearson's r** | -0.99749 | | |
| **Adj. R-Square** | 0.99248 | | |
| | | Value | Standard Error |
| **B** | Intercept | 9.87971 | 0.09307 |

| | | | |
|---|---|---|---|
| **B** | Slope | -0.01088 | 5.46E-04 |
| **LoD** | -25.66268 | | |

Table S7: Current Densities at 1000 s for biosensors prepared using WT cells cultured in SCD and characterised using EmStat Pico in horizontal assembly.

| [Cu2+] / uM | WT (SCD-Ura) | SD |
|---|---|---|
| **0** | 9.71425 | 0.09362 |
| **100** | 8.40751 | 0.15001 |
| **200** | 7.64279 | 0.23954 |
| **300** | 6.51814 | 0.16621 |

Table S8: Linear fitting and LoD calculations for current densities obtained at 1000 s for biosensors prepared using WT cells cultured in SCD and characterised using EmStat Pico in horizontal assembly.

| Fitting results WT-SCD minus Ura | | | |
|---|---|---|---|
| **Equation** | y = a + b*x | | |
| **Weight** | Instrumental | | |
| **Residual Sum of Squares** | 2.20749 | | |
| **Pearson's r** | -0.99647 | | |
| **Adj. R-Square** | 0.98941 | | |
| | | Value | Standard Error |
| **B** | Intercept | 9.6688 | 0.09148 |
| **B** | Slope | -0.0107 | 6.35E-04 |
| **LoD** | -25.74484 | | |

Table S9: Comparison of current densities for biosensors prepared using WT cells cultured in YPD and characterised using EmStat Pico in horizontal and vertical assemblies.

| [Cu2+] / uM | WT (YPD) Horizon-tal | SD | WT (YPD) Verti-cal | SD | Fold increase in WT (YPD) horizontal w.r.t WT (YPD) vertical |
|---|---|---|---|---|---|
| **0** | 9.91906 | 0.08635 | 0.90874 | 0.02342 | 10.91517926 |
| **100** | 8.53799 | 0.23961 | 0.37728 | 0.01594 | 22.63038062 |
| **200** | 7.65254 | 0.08472 | | | |
| **300** | 6.75335 | 0.15035 | | | |

Table S10: Comparison of current densities for biosensors prepared using WT cells cultured in SCD and YPD and characterised using EmStat Pico in horizontal and vertical assemblies, respectively.

| [Cu2+] / uM | WT (SCD-Ura) Horizontal | SD | WT (YPD) Vertical | SD | Fold increase in WT (SCD-Ura) Horizontal w.r.t WT (YPD) Vertical |
|---|---|---|---|---|---|
| **0** | 9.71425 | 0.09362 | 0.90874 | 0.02342 | 10.68980126 |
| **100** | 8.40751 | 0.15001 | 0.37728 | 0.01594 | 22.28453668 |
| **200** | 7.64279 | 0.23954 | | | |
| **300** | 6.51814 | 0.16621 | | | |

Table S11: Current Densities at 1000 s for biosensors prepared using C1 cells cultured in SCD-Ura and characterised using EmStat Pico in horizontal assembly.

| [Cu2+] / uM | C1 | SD |
|---|---|---|
| **0** | 7.17079 | 0.12043 |
| **100** | 6.58156 | 0.14251 |
| **200** | 5.86118 | 0.05938 |
| **300** | 4.96542 | 0.14797 |

Table S12: Linear fitting and LoD calculations for current densities obtained at 1000 s for biosensors prepared using C1 cells cultured in SCD-Ura and characterised using EmStat Pico in horizontal assembly.

| Fitting Results C1 | | | |
|---|---|---|---|
| **Equation** | y = a + b*x | | |
| **Weight** | Instrumental | | |
| **Residual Sum of Squares** | 1.8734 | | |
| **Pearson's r** | -0.9943 | | |
| **Adj. R-Square** | 0.9829 | | |
| | | Value | Standard Error |
| **B** | Intercept | 7.23963 | 0.1012 |
| **B** | Slope | -0.0071 | 5.36E-04 |
| **LoD** | -43.003 | | |

Table S13: Current Densities at 1000 s for biosensors prepared using C2 cells cultured in SCD-Ura and characterised using EmStat Pico in horizontal assembly.

| [Cu2+] / uM | C2 | SD |
|---|---|---|
| **0** | 13.61237 | 0.12369 |
| **100** | 11.40173 | 0.3005 |
| **200** | 10.29016 | 0.25946 |
| **300** | 8.68896 | 0.29214 |

Table S14: Linear fitting and LoD calculations for current densities obtained at 1000 s for biosensors prepared using C2 cells cultured in SCD-Ura and characterised using EmStat Pico in horizontal assembly.

| Fitting results C2 | | | |
|---|---|---|---|
| **Equation** | y = a + b*x | | |
| **Weight** | Instrumental | | |
| **Residual Sum of Squares** | 3.1049 | | |
| **Pearson's r** | -0.99534 | | |
| **Adj. R-Square** | 0.98606 | | |
| | | Value | Standard Error |
| **B** | Intercept | 13.56017 | 0.14867 |
| **B** | Slope | -0.01661 | 0.00114 |
| **LoD** | -26.8519 | | |

Table S15: Comparison of current densities for biosensors prepared using C1, C2 and WT cells characterised using EmStat Pico in horizontal assemblies.

| [Cu2+] / uM | C1 | SD | C2 | SD | WT (SCD) | SD | Fold increase in C2 w.r.t C1 | Fold increase in C2 w.r.t WT (SCD) | Fold decrease in C1 w.r.t WT (SCD) |
|---|---|---|---|---|---|---|---|---|---|
| **0** | 7.17079 | 0.12043 | 13.6124 | 0.12369 | 9.71425 | 0.09362 | 1.898308276 | 1.401278534 | 0.738172273 |
| **100** | 6.58156 | 0.14251 | 11.4017 | 0.3005 | 8.40751 | 0.15001 | 1.732374999 | 1.356136359 | 0.78281917 |
| **200** | 5.86118 | 0.05938 | 10.2902 | 0.25946 | 7.64279 | 0.23954 | 1.755646474 | 1.346387903 | 0.766890102 |
| **300** | 4.96542 | 0.14797 | 8.68896 | 0.29214 | 6.51814 | 0.16621 | 1.749894269 | 1.333042862 | 0.761784804 |

Table S16: Comparison Table: Enzyme/Nanomaterial-based sensors and commercially available copper detection methods (2020 – 2025)

| No. | Method & Type | Detection Range | Response Time | Cost / Operational Complexity | Reference |
|---|---|---|---|---|---|
| 1 | **Current studies** | LOD ≥ 2.2 uM | ≤ 1000s to 2500s | Moderate: requires living cells and Web-application | [7,13] |
| 2 | **Two-component CusRS biosensor (E. coli)** | LOD ≈ 0.01 µM | Rapid; high fluorescence, instrument-free | Moderate; requires engineered bacteria and fluorescence readout | [38] |
| 3 | **Yeast-based genetic biosensor** | Switch-like: 0.5–10 µM shown | ~24 h incubation | Moderate; engineered yeast, colorimetric/olfactory outputs | [15] |
| 4 | **Microfluidic enzyme-HRP chip** | 3.91 nM – 256 µM (LOD: 0.87 nM) | Rapid (naked-eye/spectrophotometric) | Moderate; microfluidic cartridge with enzyme assay | [39] |
| 5 | **Just-Add-Water DNAzyme sensor** | Threshold ~20 µM | ≤ 15 min | Low; freeze-dried reagents, no equipment required | [40] |
| 6 | **QDs ratiometric probe** | LOD 0.36 nM | Instant (UV-assisted) | Moderate; requires UV lamp for visualization | [41] |
| 7 | **N-doped carbon nanodot dip strip (CNDs)** | Visual ≥20 µM; LOD 2.28 µM | Instant, naked-eye | Low; simple paper strip with optical readout | [42] |
| 8 | **Commercial dipstick** | ~0–47.2 µM | Immediate, color chart | Very low; consumer-grade disposable strip | [43] |
| 9 | **Commercial FSTest strips** | ~1.57–31.48 µM | ≤ 30 seconds | Very low; low-cost, portable strips | [44] |
| 10 | **Hach EZ1010 Copper Cu(II) Analyzer** | ~0.47–47.23 µM (can extend via dilution) | 10 min cycle time | High complexity; automated online analyzer with low µg/L LOD | [45] |
| 11 | **Sigma-Aldrich MQuant® Copper Test Strips** | Semi-quantitative: ~157–4722 µM | Immediate (dip & read) | Very low cost; simple colorimetric strips for rough screening | [46] |

## Reference


38. Fu, Y.; Li, J.; Wang, J.; Wang, E.; Fang, X. Development of a two component system based biosensor with high sensitivity for the detection of copper ions. *Commun. Biol.* **2024**, *7*, 1–10, https://doi.org/10.1038/s42003-024-07112-6.
39. Yin, B.; Wan, X.; Qian, C.; Sohan, A.S.M.M.F.; Zhou, T.; Yue, W. Enzyme Method-Based Microfluidic Chip for the Rapid Detection of Copper Ions. *Micromachines* **2021**, *12*, 1380, https://doi.org/10.3390/mi12111380.
40. Lucci, T.J.; Neufarth, A.; Gaillard, J.-F.; Lucks, J.B. A Sensor for Detecting Aqueous $Cu^{2+}$ That Functions in a Just-Add-Water Format. *ACS Omega* **2024**, *10*, 1188–1197, https://doi.org/10.1021/acsomega.4c08751.
41. Bendicho, C.; Lavilla, I.; Pena-Pereira, F.; de la Calle, I.; Romero, V. Nanomaterial-Integrated Cellulose Platforms for Optical Sensing of Trace Metals and Anionic Species in the Environment. *Sensors* **2021**, *21*, 604, https://doi.org/10.3390/s21020604
42. Nidhisha; Kizhakayil, R.N. Onsite naked-eye detection and quantification of Cu(ii) ions in drinking water using N-doped carbon nanodots. *Mater. Adv.* **2025**, *6*, 3678–3685, https://doi.org/10.1039/d5ma00068h.
43. LaMotte Europe. Insta Test Copper and Iron Test Strip. Available online: https://www.lamotte-europe.com/products/pool-and-spa/test-strips/insta-test-copper-and-iron-test-strip-kit/en (accessed on 22 July 2025)
44. FStest. Heavy Metal Copper(Cu) Test Strip for Water. Available online: https://www.fstestcorp.com/products-detail/id-118.html (accessed on 22 July 2025)
45. EZ Series Copper Analysers. EZ1010 Copper. Available online: https://my.hach.com/ez-series-analysers/ez-series-copper-analysers/family?productCategoryId=59429629671&utm (accessed on 22 July 2025).
46. MeRcK. Copper Test. Available online: https://www.sigmaaldrich.com/IT/en/product/mm/110003?utm= (accessed on 22 July 2025).
